# Deaths of infants subject to forensic autopsy in Estonia from 2001 to 2005: what can we learn from additional information?

**DOI:** 10.1186/1478-7954-8-27

**Published:** 2010-10-05

**Authors:** Katrin Lang, Kersti Pärna, Andrej M Grjibovski, Marika M Väli

**Affiliations:** 1 Department of Public Health, University of Tartu, Tartu, Estonia; 2 Department of Infectious Diseases Epidemiology, Norwegian Institute of Public Health, Oslo, Norway; 3 Institute of Community Medicine, University of Tromsø, Tromsø, Norway; 4 International School of Public Health, Northern State Medical University, Arkhangelsk, Russia; 5 Institute of Pathological Anatomy and Forensic Medicine, University of Tartu, Tartu, Estonia; 6 Estonian Forensic Science Institute, Tallinn, Estonia

## Abstract

**Background:**

Deaths from childhood injury are a public health problem worldwide. A relatively high proportion of child deaths of undetermined manner in Estonia raises concerns about potential underestimation of intentional deaths, especially in infants. This suggests that more information on the circumstances surrounding death is needed to establish the manner of death correctly and, more importantly, to prevent these deaths. The objective of this study was to detect, describe, and analyze the circumstances around deaths of infants subject to forensic autopsy in Estonia to reveal hidden cases of child abuse and more accurately determine causes of death.

**Methods:**

Study cases included all infant deaths in Estonia from 2001 to 2005 subject to forensic autopsy at the Estonian Bureau of Forensic Medicine. Additional information was obtained from a series of visits to general practitioners, including characteristics of infant health, family composition, parents' education and employment, living conditions, and circumstances around death as perceived by medical staff in charge of outpatient services for these families.

**Results:**

The total number of infant deaths in Estonia between 2001 and 2005 subject to forensic autopsy was 98, with 40 (40.8%) deaths attributed to a disease and 58 deaths (59.2%) resulting from injury. Elements of child abuse were involved in as many as 57.7% (95% CI 46.9-68.1) of the deaths for which medical records were available (n = 90). At death, the majority of these cases were registered as diseases or deaths from unintentional injury. Average annual mortality from external causes in Estonian infants, 2001-2005, previously reported by us as 88.1 per 100,000 (95% CI 68.1-113.6) would decrease to 41.0 (95% CI 26.9-57.8).

Many infants in the studied group had faced multiple threats and were living in poor hygienic conditions. In a number of cases, they were left alone or looked after by older siblings. Parents' alcohol abuse played an important role in a considerable number of cases.

**Conclusions:**

Using additional sources of information revealed new information about child abuse not reflected in the cause of death diagnosis. Effective interventions aimed at parent education and improved follow-up of children by medical staff may reduce mortality from external causes among Estonian infants by more than half.

## Background

Deaths from childhood injury are a public health problem worldwide. The relatively high rates and cause distribution of infant deaths is of concern in many countries. In Scotland, Pearson and Stone [1] found that infant deaths constituted almost one-quarter of all injury deaths among children aged 0-14 years from 2002 to 2006. Regarding types of injury deaths, in England and Wales, infants have the highest risk to die from homicide compared to children in other age groups [2]. Similarly, in the United States, children under 2 years of age accounted for one-quarter of all homicides among children 0-14 years of age [3].

In Estonia in 2005, the crude death rate from external causes of injury and poisoning among infants was 85.1 per 100,000 children, which is seven times higher than the European Union average of 12.8 per 100,000 [4]. In our previous paper, we found that in Estonia, as in other countries, the rate of injury deaths in infants is the highest among all children in the age group 0-14 years [5].

The ill treatment and neglect of children in Estonia were among the concerns raised by the UN Committee on the Rights of the Child [6] visiting Estonia in 2004. The committee was concerned about "the insufficient information on and awareness of ill treatment and abuse of children within the family, in schools and in institutions, as well as of domestic violence and its impact on children". According to our previous study [5], the high proportion of deaths of undetermined manner raises concerns about potential underestimation of intentional deaths, especially in infants, suggesting that more information on the circumstances surrounding death is needed to establish the manner of death correctly and, more importantly, to prevent these deaths. Infant health care in Estonia is carried out by general practitioners (GPs) and nurses who perform home visits during the child's first week of life and at the practitioner's office at 2 weeks of age. From the age of 1 month, the mother should visit the child's GP or nurse every month [7].

A study from the US from 1979 to 1988 revealed that 85% of child abuse and neglect deaths were recorded as due to other causes [8]. Underreporting of child abuse deaths is a common finding also in current studies that have looked at causes of children's deaths [9-11].

In the current study, we searched for more detailed information on how the data are generated for cause of death determination. Here, we present information collected from medical records of the deceased infants and staff interviews, revealing important facts such as elements of neglect. Such acts of omission have been found to cause underestimation of child injury deaths, particularly among infants with specific sociodemographic characteristics, such as African Americans and those residing in urban settings at the time of death, although the effect estimates were imprecise [12].

The aim of the study is to detect, describe, and analyze the circumstances around deaths of infants subject to forensic autopsy in Estonia to reveal hidden cases of child abuse and more accurately determine causes of death.

## Methods

Study cases included all infant deaths in Estonia from 2001 to 2005 subject to forensic autopsy at the Estonian Bureau of Forensic Medicine (as of 2008, the Estonian Forensic Science Institute). This is a subsample of a larger study that described intents and causes of death among children aged 0-14 years. The methods of data collection have been presented in detail previously [5].

Forensic medical autopsy in Estonia is conducted if there is evidence or suspicion of a crime, when the death is caused by external factors but no crime is suspected, in cases of postmortem decomposition, or if the identity of the deceased is unknown [13]. Thus, all infant injury deaths are subject to forensic autopsy.

For the current study, additional information was obtained by a series of visits to GPs. Information that was sought included characteristics of infant health, such as acute and chronic illnesses recorded, but also alcohol abuse in the family and circumstances around death as perceived by medical staff in charge of outpatient services for these families. This information was retrieved from outpatient records, and in most cases was complemented by interviews with GPs and/or nurses providing outpatient care for these families and recorded on data collection forms.

Data were computerized, and the section containing data required for identification of cases was sent to the Statistical Office of Estonia, which traced the underlying cause and manner of death, allowing for comparisons with national death statistics.

The system of death registration in Estonia has been described elsewhere [14]. In addition, the Statistical Office of Estonia provided data on maternal education of the study subjects.

Data were analyzed using Stata 9.2 (STATA Corp, TX, USA). Descriptive statistics were calculated, including odds ratios (OR) with 95% confidence intervals (95%CI) comparing the characteristics of infants dying from injuries vs those dying from diseases. A combined indicator of child abuse was generated, reflecting the presence of physical abuse and/or child neglect. Using this indicator, a proportion of preventable deaths was calculated, considering a definition of preventable death by Rimsza et al [15]: "if an individual or the community could reasonably have done something that would have changed the circumstances that led to the child's death". Physical child abuse was defined as physical aggression directed at a child by an adult, and child neglect was defined as the situation where the responsible adult fails to adequately provide for various needs, including physical (failure to provide adequate food, clothing, or hygiene), emotional (failure to provide nurturing or affection), or educational (failure to enroll a child in school) [16].

The study was approved by the Ethics Review Committee on Human Research at the University of Tartu, Estonia (protocol no 101/2).

## Results

The total number of infant deaths in Estonia between 2001 and 2005 subject to forensic autopsy was 98, or 22.1% of the total number of infant deaths (n = 444) for the given time period. Boys constituted 58.2% (n = 57) of the cases.

**Figure 1:**
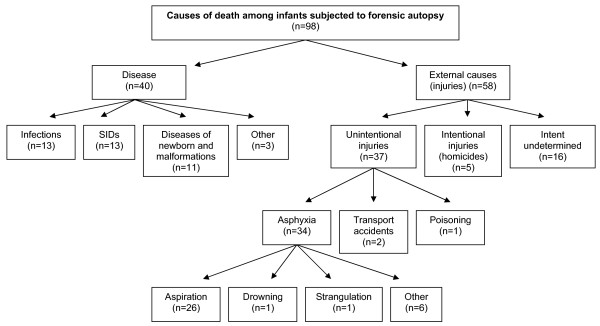
**Distribution of intents of death among infants subjected to forensic autopsy in Estonia from 2001 to 2005**.

Among 98 autopsied infants, 40 (40.8%) deaths were attributed to a disease and 58 (59.2%) were the result of injury (Figure 1). The distribution of causes of death from diseases was as follows: 32.5% died from infectious diseases; 32.5% died from sudden infant death syndrome; 27.5% died from congenital malformations and diseases of the newborn; and 7.5% died from other diseases (epilepsy, cardiac arrest, and subarachnoid hemorrhage). Among injury deaths, 63.8% were attributed to unintentional injuries, 8.6% to intentional injuries, and 27.6% to injury deaths of undetermined intent. Among unintentional injury deaths, asphyxia, mainly caused by aspiration, was the most common cause of death (91.9%).

Medical documentation was available for 90 cases. For the remaining eight cases, intents of death were the following: intent of death undetermined (6), unintentional injuries (1), and disease (1) as recorded in the Statistical Office of Estonia.

Basic characteristics of the study cases and odds ratios for exposure are presented in Table 1.

**Table 1:** Characteristics of study cases, numbers (No), percentages, and odds ratios (OR) with 95% confidence intervals (95%CI) comparing infants dying from injuries vs dying from diseases

	Total	Died from diseases	Died from injuries	p-value	OR (95%CI)
**Characteristic**	**No**	**No (%)**	**No (%)**		
**Age of infant at death, months**					
**<3**	41	19 (48.7)	22 (43.1)		1
**3-5**	33	12 (30.8)	21 (41.2)		1.51 (0.59-3.86)
≥**6**	16	8 (20.5)	8 (15.7)	0.58	0.86 (0.27-2.74)
**Birth order**					
**1**	20	11 (28.2)	9 (17.7)		1
**2**	27	12 (30.8)	15 (29.4)		1.53 (0.48-4.89)
**≥3**	22	11 (28.2)	11 (21.5)		1.22 (0.36-4.12)
**not known**	21	5 (12.8)	16 (31.4)	0.19	3.91 (1.03-14.87)
**Place of death**					
**home**	78	36 (92.3)	42 (82.4)		1
**hospital**	8	2 (5.1)	6 (11.7)		2.57 (0.49-13.54)
**other**	4	1 (2.6)	3 (5.9)	0.39	2.57 (0.26-25.82)
**Mothers education**					
**basic**	50	22 (56.4)	28 (54.9)		1
**secondary/higher**	40	17 (43.6)	23 (43.1)	0.89	1.03 (0.68-1.57)
**Alcohol abuse in family**					
**no**	68	28 (78.4)	40 (71.8)		1
**yes**	22	11 (21.6)	11 (28.2)	0.47	1.43 (0.54-5.75)

There were no significant differences in these characteristics between infants dying from diseases and those dying from injuries. Most infants died during the first half-year of life. Regarding the place of death, the majority of infants died at home. Mothers' education was low in general, with more than half of mothers having basic education. Only one mother had higher education.

The odds of dying from injuries were higher for infants between ages 3 and 5 months, those who were second/higher born or whose birth order was not known, and those with alcohol abuse in the family, although the results did not reach the level of statistical significance.

Elements of child abuse, such as physical abuse or neglect, that could be classified as preventable deaths according to the definition by Rimsza et al [15] were involved in as many as 52 (57.7%) of the deaths for which medical records were available (Table 2). Preventable deaths were distributed similarly between deaths from diseases and deaths from injuries.

**Table 2:** Distribution of manners of death and preventable deaths.

Manner of death	Number of cases	Death preventable			
		**number**	**% (95% CI)**		
**Disease**	39	25	64.1 (47.2-78.8)		
**Injury**	51	27	52.9 (38.5-67.1)		
**Unintentional injury**	36	20	55.5 (38.1-72.1)		
**Homicide**	5	1	20.0 (0.5-71.6)		
**Undetermined**	10	6	60.0 (26.2-87.8)		
**Total**	90	52	57.7 (46.9-68.1)		

Information from Table 2 was used to calculate the expected injury mortality rate among infants in Estonia. Average annual mortality from external causes in Estonian infants, 2001-2005, previously reported by us as 88.1 per 100,000 (95% CI 68.1-113.6) would decrease to 41.0 per 100,000 (95% CI 26.9-57.8).

Regarding illnesses, more than one-third of infants (41.1%) suffered from one or more acute or chronic medical conditions. The most frequent diseases were diarrhea, acute illnesses of the upper respiratory tract, and hypotrophy. Thirteen deaths (14.4%) were attributed to infectious diseases.

A selection of study cases where information collected during the study was not reflected in the cause/manner of death is presented in Table 3.

**Table 3:** Examples of study cases where information collected during the study is not reflected in the cause/manner of death.

Sex	Age (months)	Cause of death	Manner of death	Description of the family/household/circumstances
M	5	X59: Exposure to unspecified factor	not established	The child unattended, mother unemployed, father with criminal record
M	0	P52: Intracranial nontraumatic haemorrhage of fetus and newborn	disease	Mother HIV+, living with her parents, wanted to terminate her pregnancy at 35 weeks and was only then registered for pregnancy followup
F	4	R 95: Sudden infant death syndrome	disease	Single 18-year-old mother, HIV+, narcotics use.
M	3	R 95: Sudden infant death syndrome	disease	On admission to hospital, the child was dirty with filthy clothes, no diaper. At autopsy, bruising and abrasion of the forehead was detected.
M	3	W 78: inhalation of gastric contents	accident	The parents were drug addicts, lived in a shared flat with a number of people.
M	1	J 15: Bacterial pneumonia, not elsewhere classified	disease	On several occasions, the mother was drinking alcohol when home alone with the child. According to a friend, she smothered the child with a pillow. She was later accused of beating the next child and was imprisoned.
F	10	A09: Diarrhea and gastroenteritis of presumed infectious origin	disease	Mother was HIV+ and a drug addict. According to the forensic autopsy protocol, she could have killed the baby (not enough evidence to state that in the diagnosis).
M	4	R 95: sudden infant death syndrome	disease	Mother had lues (the child had congenital lues) and a psychiatric disorder. Father had alcohol problems. The child had convulsions at home, the ambulance was called, and on arrival, they found the child dead.
F	2	J 15: Bacterial pneumonia, not elsewhere classified	disease	Mother had 4 older children. Lived in a shabby household. Left the child who later died under the care of older children, who fed her, and the baby aspirated food.
M	5	W 78: inhalation of gastric contents	accident	The child was born at home. Mother was a drug addict. Both parents had alcohol problems. The household was shabby and dirty. Older children had been taken to care (removed from parents).
M	4	G00: Bacterial meningitis, not elsewhere classified	disease	The child was hit in the face, had a broken rib, was at home with mother's partner.

## Discussion

This study is among the first in the Baltic states to use the data on all infant deaths subject to forensic autopsy together with supplementary information from medical records and interviews with GPs or nurses. However, several limitations of the study should be mentioned. The sample consisted of 98 cases, which is a small number, especially for subgroup analysis. Yet it accumulates all eligible cases from the whole of Estonia over a five-year period.

Medical documentation and/or additional information from the GP or nurse was not available for eight cases. All of these children died before the second month of life. They were most probably not registered with a GP, although registering with a GP during the first month of life is part of routine medical practice in Estonia. Considering the fact that six of them were deaths with manner of death undetermined, we may speculate that having been able to collect additional information regarding these cases would have contributed to underestimation of child abuse deaths.

The fact that some evidence of child abuse was available for the surprisingly high proportion of deaths classified as disease, unintentional injury, or death of undetermined manner, suggests that the Estonian national mortality statistics underestimate infant deaths that might be associated with abuse and neglect.

Similar findings have been reported by Crume et al, who detected that half of fatalities associated with child maltreatment in the age group 0-16 years were not ascertained by the death certificates in Colorado in 1990-1998 [12]. They compared data collected by a multidisciplinary child fatality review team with vital records for children aged 0-16 years and concluded that only half of children who died as a result of maltreatment had death certificates that contained information on maltreatment. Also, they found that death resulting from violent causes such as shaking, striking, etc., were more likely to be ascertained than those that involved acts of omission. In our study, similarly, there were a number of cases in which additional information revealed forms of neglect (infants were left alone or looked after only by older siblings) not considered in the cause of death diagnosis.

Regarding the types of diseases recorded in the medical history, hypotrophy, diarrhea, and acute upper respiratory illnesses may at least partly be attributed to poor living conditions, lack of care, or lack of knowledge by parents. In Estonia, it is primarily the mother who liaises with medical staff. In cases of acute illness, mothers can call the GP practice and register for a home visit, but less educated and young mothers may not have enough knowledge to recognize acute illness or detect its severity. Recent studies in Estonia [17,18] have shown that low maternal education increases injury mortality among infants and toddlers. We were able to show low maternal education among the study group. Tiikaja et al [17] were also able to detect higher risk of injury deaths with increasing birth order, which we were not able to show, perhaps due to the small sample and missing data on birth order.

Using additional sources of information revealed new information about child abuse not reflected in the cause of death diagnosis. Elements of child abuse were involved in nearly two-thirds of the deaths. The official cause of death statistics in Estonia underreport child deaths caused by child abuse. Incorporating this information in diagnosing the cause of death and investigating the circumstances of child injury deaths in detail would improve reporting causes of death in infants.

Child mortality review teams have been successful in determining preventable deaths in other countries [19]. This practice has not yet been implemented in Estonia. Recently, there have been some activities in parent education in Estonia targeting parents at risk. The target groups of the European Dimension in Parent Education project in Estonia, funded with support from the European Commission from 2007 to 2009, included economically or socially disadvantaged groups and offeredparent education and home visits. Based on the Amnesty International Report 2004 for Estonia, the recommendations included explicit prohibition of corporal punishment, implementation of measures to prevent physical and mental violence, and the establishment of effective mechanisms to receive, monitor, and investigate complaints [6].

The results of the study suggest that effective interventions aimed at parent education and improved follow-up of children by medical staff may reduce mortality from external causes in Estonian infants by more than half.

## Competing interests

The authors declare that they have no competing interests.

## Authors' contributions

This study was devised by KL and MV. KL and MV organized and undertook the field visits and abstraction of information from medical records and interviews with staff. KL arranged and carried out the linkage to the data of the Statistical Office of Estonia. KL, AG, and KP analyzed the data. All authors drafted the paper. The interpretation of the results and the final draft of the paper involved input from all four authors. All authors have read and approved the final manuscript.
